# Ultrafast electron diffraction optimized for studying structural dynamics in thin films and monolayers

**DOI:** 10.1063/1.4949538

**Published:** 2016-05-12

**Authors:** D. S. Badali, R. Y. N. Gengler, R. J. D. Miller

**Affiliations:** 1 Max Planck Institute for the Structure and Dynamics of Matter, Hamburg Centre for Ultrafast Imaging, Department of Physics, University of Hamburg, Luruper Chaussee 149, Hamburg 22761, Germany; 2 Departments of Chemistry and Physics, University of Toronto, 80 St. George Street, Toronto M5S 1H6, Canada

## Abstract

A compact electron source specifically designed for time-resolved diffraction studies of free-standing thin films and monolayers is presented here. The sensitivity to thin samples is achieved by extending the established technique of ultrafast electron diffraction to the “medium” energy regime (1–10 kV). An extremely compact design, in combination with low bunch charges, allows for high quality diffraction in a lensless geometry. The measured and simulated characteristics of the experimental system reveal sub-picosecond temporal resolution, while demonstrating the ability to produce high quality diffraction patterns from atomically thin samples.

## I. INTRODUCTION

Ultrafast structural dynamics is a new and rapidly growing research field, driven by both the desire to witness atomic motions in real-time and the emergence of technology with this capability. One of the most promising of such technologies is ultrafast electron diffraction (UED), a table-top pump-probe technique that exploits the sub-angstrom wavelength of accelerated electron pulses to obtain the temporal and spatial resolution required to measure transient structural dynamics.^1–3^ Since the seminal work of Siwick *et al*. in 2003 (Ref. 4), performed using 30 keV electron bunches produced in a “compact” electron gun assembly, instrumental development has primarily been focused on the fabrication of UED setups with higher acceleration voltages. Most modern machines fall into two categories, either being table-top setups with energies in the 30–200 keV range,^4–11^ or 5–7 MeV radio-frequency driven linear accelerator sources.^12–18^

The reason for the push into the high-energy regime is that the energy of the probe electrons ultimately determines what thickness of material the setup will be capable of studying. In general, higher energy gives UED access to thicker samples, since the mean free path of electrons increases significantly with energy.^19^ In fact, this has been the guiding principle behind the recent development of high-energy UED sources: toward the ultimate goal of studying dynamics in relatively thick (100 nm–1 *μ*m) protein crystals.^20^

At the other extreme, low-energy electrons scatter more efficiently from thinner samples,^21^ a fact which is of particular interest to the materials science community. The rise of two-dimensional materials, such as graphene^22^ and the plethora of others,^23–27^ has revived interest in the study of monolayers and thin films. In fact, such materials are touted as having the potential to revolutionize the future of many applied fields, such as electronics, energy storage, and optics. Although the number of known materials possessing interesting natural or synthetic low-dimensionality properties is perpetually growing,^23^ the development of techniques used to investigate the structure of these materials is fairly stagnant; in fact, the standard techniques have hardly changed in the last couple of decades, with the latest advancement being the development of scanning probe microscopies such as scanning tunneling microscopy (STM)^28^ and atomic force microscopy (AFM)^29^ in the 1980s.

With this in mind, we advocate that the field of thin films would certainly be enriched by the availability of an ultrafast structural probe. It is to this end that we designed and built an ultrafast electron diffractometer, which is specifically optimized for studying free-standing thin films and monolayers. As will be discussed below, this optimization comes from choosing to work in the “medium” energy range of 1–10 kV. Such acceleration energies have been used in previous UED machines,^30,31^ although exclusively in reflection mode. While this provides sensitivity to the surfaces of bulk samples, it is not practical to study free-standing thin films. Several setups have pushed into the true low-energy regime (<500 eV) and have had success using tip-based sources to measure static^32^ and time-resolved diffraction^33^ from free-standing thin films. While this is a similar approach to what we present here, our work distinguishes itself by the simplicity of the instrumental design, which is achieved by employing a planar photocathode and a lensless system.

## II. DESIGN PRINCIPLES

There is a well-known^34,35^ and inherent challenge to working with short electron pulses: because electrons are negatively charged, dense bunches of them tend to expand due to space-charge effects. This significantly degrades the temporal resolution of the experiment. This effect is particularly pronounced when working in the low/medium energy regime, because both longitudinal and transverse beam growth due to space-charge effects scales as ${\left(1-\beta \right)}^{-3/2}$ (Ref. 36), where *β* is the ratio of the electrons' speed to the speed of light. Since *β* is proportional to the square root of the acceleration voltage, lower energy electrons suffer significantly from unwanted space-charge effects. Because of this, adapting the UED technique for low/medium energy electrons to study thin films requires some particular design considerations that are absent in current state-of-the-art setups.

While space-charge effects are typically managed by scaling-up the compact electron gun design concept and including electron optics to compress the pulses,^37–41^ this paper takes the exact opposite approach: by scaling-down the electron gun and removing all electron optics. The remainder of the article will demonstrate a compact, lensless, medium-energy setup with sub-picosecond temporal resolution and sensitivity to films with thicknesses <10 nm.

**FIG. 1.**
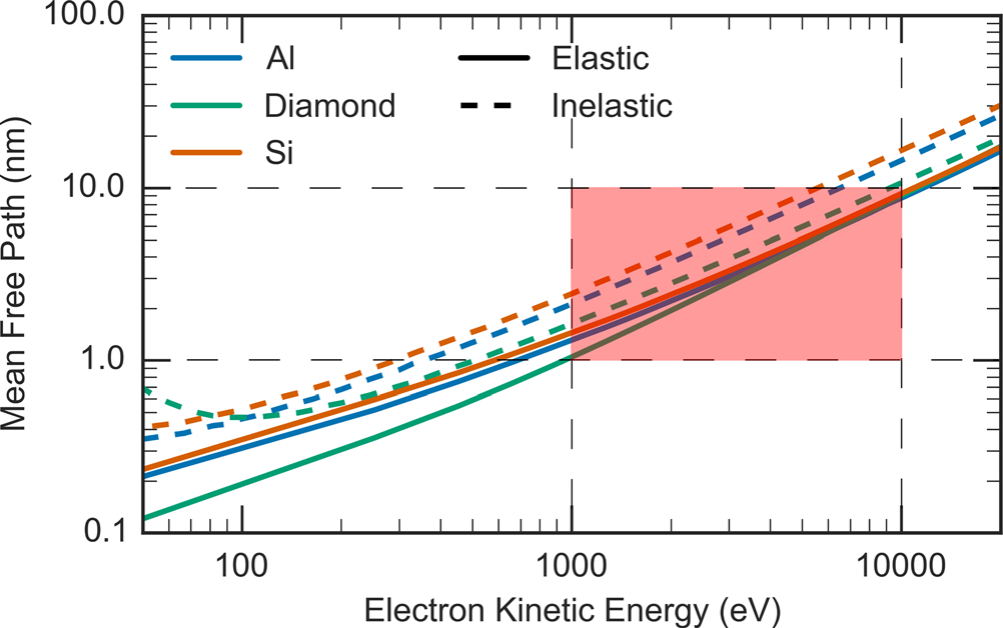
Comparison of the energy dependence of the elastic (solid lines) and inelastic (dashed lines) mean free path of several materials. The shaded area indicates the operating region for ultrafast electron diffraction of free-standing thin films and monolayers. The mean free paths were calculated using elastic scattering cross sections from Ref. 79, inelastic scattering cross sections from Ref. 80, and number densities from Ref. 81.

The first choice when designing a UED setup is the acceleration voltage of the electron gun, as this will dictate all subsequent design choices. As stated in the introduction, in order to be sensitive to thin films and monolayers the machine presented here must use electrons with energy at the lower end of the spectrum. This is also the motivation behind the surface sensitive technique low-energy electron diffraction (LEED). However, the low energies used in LEED (<500 eV) are not optimum for performing transmission UED experiments on samples other than single monolayers. To see this, consider the following: in general, the electron energy should be chosen such that the mean free path Λ(E) satisfies Λ(E)≥d, where *d* is the sample thickness. This ensures that each electron undergoes at most one elastic scattering event as it traverses the sample (on average). However, if the energy is too large, most electrons do not scatter at all, which dramatically reduces the efficiency of the experiment. As such, it is preferable to choose the energy such that Λ(E)≈d so that most electrons scatter elastically once. To quantify the acceptable energy range, Fig. 1 plots the elastic and inelastic mean free paths for several common materials. In reference to these data, to study films of 1–10 nm in transmission mode, this reasoning implies that the preferable energy should be in the range of 1–10 kV.

**FIG. 2.**
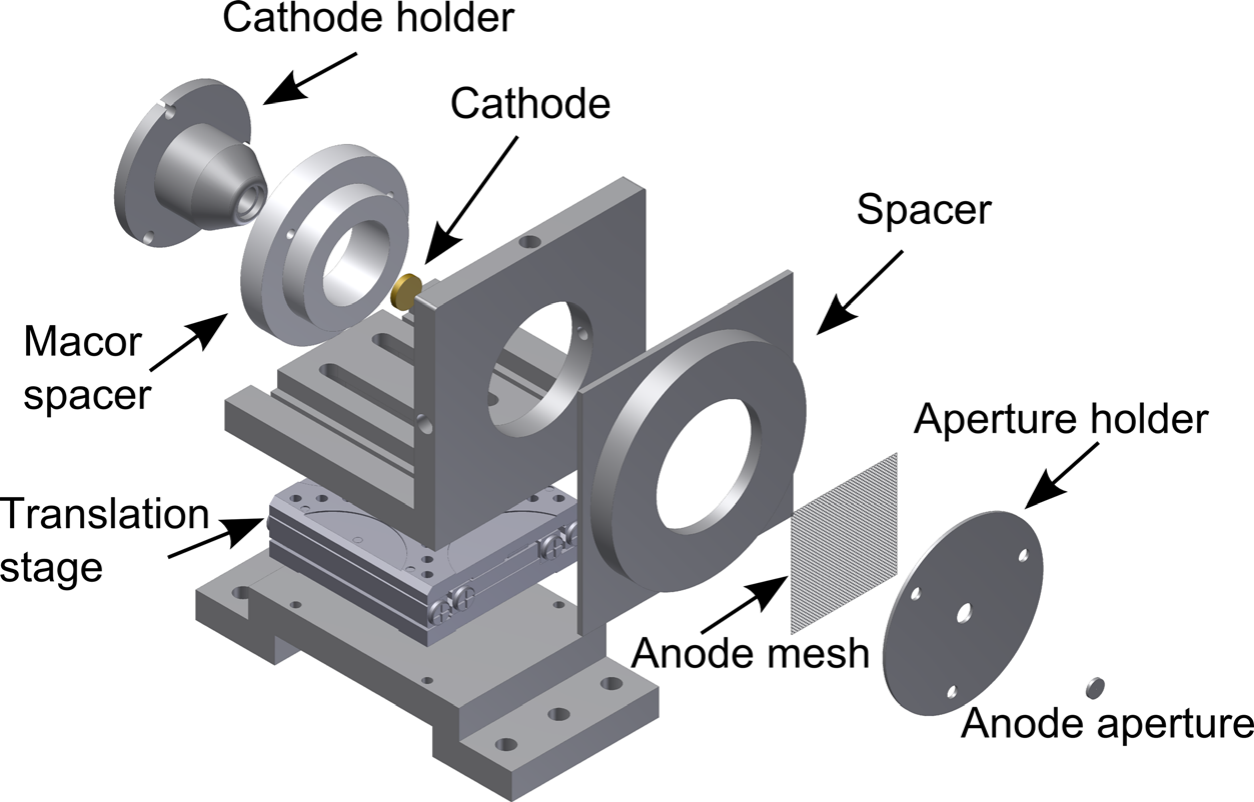
Simplified exploded drawing of the ultrafast electron gun.

Having decided on the energy range which is most appropriate for studying thin films and monolayers, we can proceed with the presentation of the design of the UED setup, which is illustrated in Fig. 2: the photocathode was chosen to be a planar metallic film illuminated from the back by ultraviolet light. While this is known to produce slightly inferior beam properties compared to front-illuminated photocathodes^42^ or tip-emitters,^43–47^ the small cathode-anode spacing of the electron gun design (presented in the following paragraphs) inhibits front- or side-illumination. The photocathode consisted of a 20 nm thick gold film, which had been thermally evaporated on a quartz plate coated with a 3 nm thick chromium layer.

The photocathode assembly was mounted on a movable pedestal to allow for a variable cathode-anode distance. This unique feature was used during the conditioning of the gun to maximize the accelerating electric field while avoiding arcing. To minimize temporal broadening of the electron pulses, all dimensions were kept at the smallest mechanically feasible values. In a typical measurement, the cathode-anode separation distance was 1 mm, and the sample and anode plate were brought into direct contact. The sample-to-anode distance was therefore only limited by the anode thickness (≈0.5 mm).

Significant distortions in the electron beam shape were observed when using a traditional anode, which is simply a flat metallic plate with a small aperture in it. The inhomogeneous penetration of the accelerating electric field into the aperture is known to cause a defocusing effect,^48^ which accounts for the observed distortions. To mitigate this, the densest available mesh (2000 lines/in., Ted Pella) was mounted over a 40 *μ*m diameter aperture, resulting in the effective removal of the observed distortions. This results from the fact that the defocusing effect of each hole in the mesh is significantly less than that of a larger aperture due to the reduction in the penetration of the electric field. This observation can be quantified by appealing to an analogy with geometric optics: for a negative lens with a focal length of *f*, the induced divergence is θ=y/|f|, where *y* is the aperture of the lens. From this equation, we see that from going from an aperture anode to a mesh anode the beam divergence reduces by a factor of

$$\frac{\theta_{\text{aperture}}}{\theta_{\text{mesh}}}=\frac{y_{\text{aperture}}}{\left|f_{\text{aperture}}\right|}\times \frac{\left|f_{\text{mesh}}\right|}{y_{\text{mesh}}}.$$ (1)

Here, we note that, even though the mesh has many holes, $\theta_{\text{mesh}}$ represents the angle of the maximally divergent ray. The focal length of the weak negative lens formed by the penetration of the accelerating electric field is approximately independent of the aperture size,^49^ and so this expression simplifies to $\theta_{\text{aperture}}/\theta_{\text{mesh}}=y_{\text{aperture}}/y_{\text{mesh}}$. Using the experimental parameters for our experimental setup, this reveals that the divergence is reduced by a factor of 5 when going from an aperture to a mesh anode. The induced divergence of ≈0.05° due to the mesh anode is negligible relative to the broadening due to space-change effects. It is worth noting that our setup's ability to optimize the cathode-anode separation distance for any given electron energy reduces the defocusing effect for the energy-scale considered here relative to high-energy electrons.

A distinguishing feature of the instrument described here is the absence of active collimating and focusing elements such as electrostatic or electromagnetic lenses (either before and or after sample). These are components that are staples in traditional UED setups. The lensless construction is a consequence of many design choices: since the planar gold cathode emits electrons with minimal divergence and using a mesh anode further minimizes defocusing, the electron beam reaches the sample with nearly parallel momentum. The omission of all lenses dramatically reduces the electron gun-to-sample electron flight path and therefore significantly combats the degradation of the temporal resolution of the experiment due to longitudinal space-charge broadening. It is worth noting that omitting a lens between the cathode and the sample to reduce space-charge is not novel (see, for example, Refs. 9–11). However, most UED machines compensate for this by using a lens after the sample to focus the electrons onto the detector. The UED setup presented here is unique in its completely lensless design.

## III. SIMULATED AND EXPERIMENTAL PERFORMANCE

**FIG. 3.**
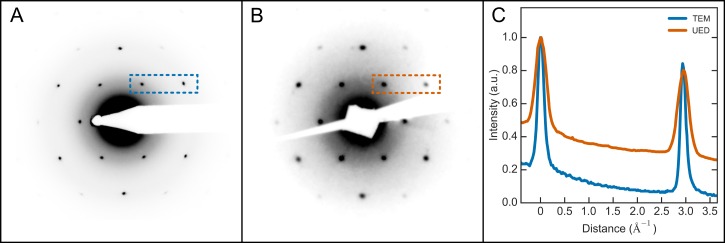
Comparison of the diffraction pattern of graphene on a copper mesh coated with a lacey carbon film measured with (a) a transmission electron microscope (TEM) at 80 kV and (b) the ultrafast electron diffraction (UED) setup at 6 kV. The line profiles in (c) correspond to the highlighted regions in the diffraction patterns in (a) and (b). These data have been normalized to the intensity of the first order peak. The widths and locations of neighbouring Bragg peaks were used to estimate the transverse coherence length of the UED setup.

Using graphene as a prototypical thin film, Fig. 3 demonstrates the capabilities of the UED setup for measuring structural dynamics in thin films and monolayers. Fig. 3(b) shows a typical diffraction pattern from graphene mounted on a copper mesh coated with lacey carbon (Ted Pella) measured with the UED setup with an acceleration voltage of 6 kV with the experimental parameters listed in Table I. To provide a point of reference on the quality of the diffraction, Fig. 3(a) shows a diffraction pattern from the same sample measured with a Philips CM12 transmission electron microscope (TEM) operating at 80 kV and illuminating an area of a few hundred nanometers. From visual inspection, it is evident that there is a qualitative agreement in the quality of the diffraction pattern taken with the UED setup and the TEM. To quantify this, Fig. 3(c) shows the line profiles taken from the indicated regions in the diffraction images. These data have been normalized to the intensity of the first order peak. It is clear that the TEM has superior signal-to-background. Additionally, the TEM exhibits significantly more narrow peaks, which is indicative of a higher degree of coherence (see the discussion on coherence below). However, the quality of the UED diffraction is ample for the intended experiments. For graphene, diffraction past the fourth order was observed.

It should be noted that, with a thickness of <1 nm, graphene is thinner than the ideal sample for the UED setup presented here. Although it would be more optimally studied at lower energies for higher diffraction efficiency, graphene serves to demonstrate the capabilities of the UED setup to measure adequate signals from true monolayers.

We proceeded by performing several measurements to quantify the quality of the UED setup. First, the width of the electron pulses at the sample was measured using a knife-edge technique to be ≈70 *μ*m full-width at half-maximum (FWHM). It is important to note that this is somewhat smaller than what is typical for UED experiments;^1,11,50–52^ however, since thin films and monolayers are more susceptible to surface roughness and rippling than their bulk counterparts,^53–55^ such a narrow beam is required for acceptable quality diffraction.

Although there are many characteristics that are imperative for a successful UED experiment, one of the most prominent is the transverse coherence length of the electron pulses. This parameter can be estimated from the diffraction pattern of a sample with a known structure.^56^ This analysis was performed on a typical diffraction pattern from graphene using the two peaks shown in the highlighted rectangle in Fig. 3(b). Calculating the widths of the peaks in the line profile (Fig. 3(c)) results in a coherence length of ≈2.9 nm. This value is comparable to state-of-the-art UED machines,^1^ and, since it spans several unit cells for most inorganic solids, is sufficient for UED experiments. It should be emphasized that this estimate is a lower limit of the transverse coherence length.

The other important parameter in a UED experiment is the temporal resolution of the system, which is typically limited by the pulse duration of the electron bunches. While several experimental techniques have demonstrated the ability to measure the pulse duration,^57–63^ unfortunately the extremely compact design of the medium-energy electron gun presented here precludes such measurements. Therefore, we turned to detailed particle tracing simulations using ASTRA.^64^ ASTRA is a sophisticated software suite that performs fully relativistic to non-relativistic, non-quantum simulations of particle propagation through external fields, while accounting for space-charge effects and other complex phenomena such as secondary electron emission and mirror charges at the cathode.^64^ While such simulation tools are limited in the physical effects that they model, they have become an established tool in the UED community for estimating transient electron pulse characteristics. That being said, it is important to keep in mind that the simulated results are the best estimations under the confines of ASTRA.

The input to the ASTRA simulation was the initial distribution of the electron bunch at the cathode, which was estimated from the measured properties of the probe laser beam. Spatially, the initial electron bunch was taken to be an uncorrelated, two-dimensional normal distribution with a FWHM of 27 *μ*m, identically matching the measured spatial profile of the probe laser. The initial momentum distribution was characterized by the three-step model for photoemission,^65^ which accounts for the Fermi-Dirac distribution of states in the cathode. The inputs to this calculation were the probe photon energy and the effective work function of the cathode (accounting for any lowering due to the Schottky effect). This results in a calculated initial energy spread of roughly 90 meV. Temporally, the emission of the electrons from the cathode was taken to match the pulse profile of the probe laser, and so was taken to be Gaussian with a 165 fs FWHM. This value accounts for pulse broadening in the third-harmonic generation crystals and was determined through simulations using the SNLO software.^66^ Simulations were performed for various numbers of electrons in the bunch, ranging from 10^3^ to 10^5^, and also with acceleration voltages in the range of 1–10 kV. A summary of the parameters used in the simulations can be found in Table I. These parameters were chosen to match the corresponding experimental values.

**FIG. 4.**
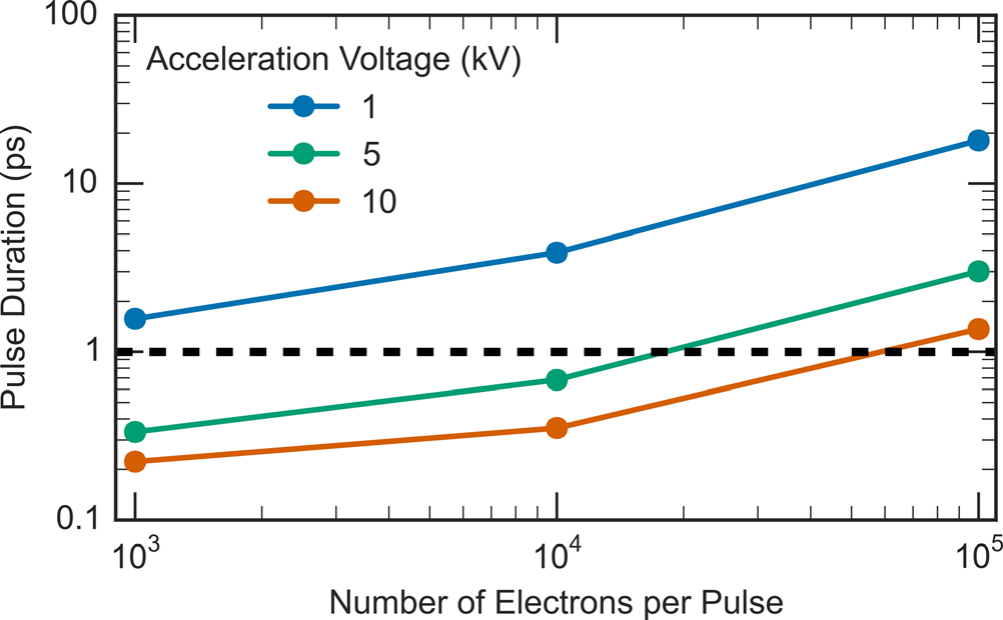
Full-width at half-maximum pulse duration of electron bunches at the sample location simulated with ASTRA. The input parameters to the simulation are listed in Table I.

The simulated FWHM pulse duration at the sample is shown in Fig. 4. It should be noted that these calculations are for a fixed acceleration gap between the cathode and the anode, with the highest realistic extraction field of 10^5 ^V/cm corresponding to the 10 kV electron curve. In our design, it is possible to adjust the gap to keep the field constant in which case the expected pulse duration is in the 300–400 fs regime at the maximum extraction field for all electron energies for electron bunches up to 10^4^ electrons. In this energy range, the quantum efficiency of conventional, low cost, microchannel plates (MCPs) is close to unity such that 10^4^ electrons is sufficient for single shot structure determination. This source brightness is essential to get reasonable signal-to-noise ratios (SNR) at the low repetition rates needed to avoid thermal artifacts. This concept is in contrast with proposals to use single electrons to avoid space-charge effects^68^ for which these extremely low brightness sources require more than ×10^5^ longer acquisition times (taking only into account the statistics for ensuring operation in the single electron limit) to achieve the same SNR. These simulations show that you do not need to go to the single electron limit to avoid space charge broadening and still retain sufficient spatial/temporal resolution to observe atomic motions on the primary time scales of structural transitions. These results reveal that it is possible to obtain sub-picosecond durations for low acceleration voltages for reasonably high electron bunch density. This is a promising result and suggests the UED setup introduced here can achieve femtosecond temporal resolution with acceleration voltages of a few kilovolts (>1 kV) in the single shot limit for small (a few nanometers) unit cell systems.

**FIG. 5.**
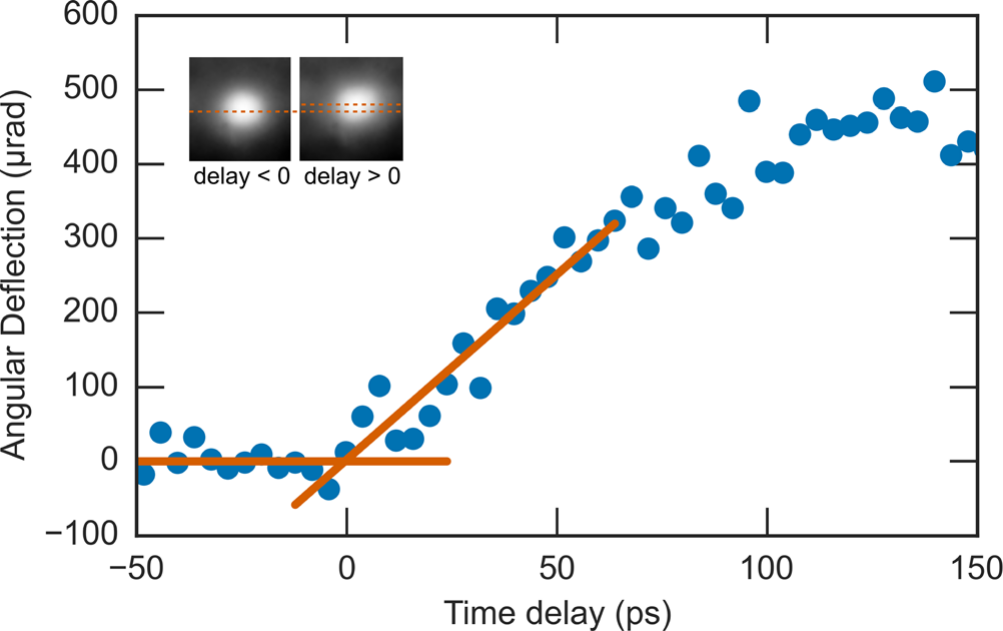
Method for finding the temporal overlap in an ultrafast electron diffraction experiment: observation of the transient deflection of the direct electron beam as a result of ultrafast plasma formation generated by irradiating a copper mesh with an intense 800 nm femtosecond laser at 4 mJ/cm^2^. The temporal overlap was identified as the crossing of two linear fits, one before laser excitation and the other for the rising edge of the plasma formation. The inset images show the electron beam before and after the arrival of the excitation laser. The centroid of each spot has been identified to highlight the deflection of the beam.

To demonstrate the ability of the medium-energy UED system to measure temporal dynamics, we measured the deflection of the electron pulses after passing through the transient electric fields produced by the plasma emitted from a laser-irradiated metal surface. This is a well-studied phenomenon^69–76^ and has become popular in the UED community for identifying the temporal overlap of the pump and probe.^76–78^ The results of these experiments are presented in Fig. 5, which shows the deflection angle of a 6 kV electron beam passing through a 300 lines/in. copper mesh irradiated by an 800 nm pump at a fluence of 4 mJ/cm^2^. The geometry of the experiment was the same as listed in Table I, resulting in an extraction field of 6 × 10^4 ^V/cm. The measured time-scale, on the order of tens of picoseconds, is in agreement with similar measurements of transient deflection due to plasma formation.^69–72^

The temporal overlap was determined by finding the intersection of two linear fits to the transient data: one before laser excitation (i.e., that background, representing zero deflection) and one for the rising edge of the electron beam deflection (see Fig. 5). Using this approach, we were able to determine the temporal overlap with approximately 1 ps accuracy, which is calculated by the propagation of the uncertainty in the linear fits.^77^

This measurement serves to both identify the temporal overlap between the pump and the probe and demonstrate the UED setup's potential for measuring transient dynamics in thin films.

## IV. CONCLUSIONS AND OUTLOOK

The experimental system presented here extends the technique of ultrafast electron diffraction to the medium-energy regime and introduces several design principles to optimize the sensitivity to free-standing thin films and monolayers. The measured and simulated characteristics of the experimental system presented here suggest it possesses sub-picosecond resolution, while demonstrating the ability to produce adequate quality diffraction patterns from atomically thin samples. These are the ingredients required to perform ultrafast electron diffraction of thin films and monolayers.

The challenge now is to develop samples that exhibit various aspects of surface reaction dynamics, catalysis, and novel two-dimensional confined structural dynamics. This class of samples will be particularly prone to thermal effects and will require relatively low repetition rates or large surface areas to enable equilibration between laser excitation events. This electron source concept will provide the needed space-time resolution and brightness for the two-dimensional exploration of structural dynamics.

## Figures and Tables

**TABLE I.** Parameters for the simulation of the ultrafast electron diffractometer using ASTRA. These values correspond to typical operating conditions for ultrafast electron diffraction experiments.

Parameter	Value
Laser pulse duration (FWHM)	165 fs
Laser spot size on the cathode (FWHM)	27 *μ*m
Photon energy	4.65 eV
Cathode effective work function^67^	4.26 eV
Initial energy spread	≈90 meV
Cathode-anode distance	1 mm
Anode thickness	0.25 mm
Anode-sample distance	0.5 mm
Anode aperture radius	20 *μ*m
Electrons per pulse	10^3^–10^5^
Charge per pulse	0.1–10 fC
Acceleration voltage	1–10 kV

## References

[1] G. Sciaini and R. J. D. Miller , “ Femtosecond electron diffraction: Heralding the era of atomically resolved dynamics,” Rep. Proj. Phys. 74, 096101 (2011). 10.1088/0034-4885/74/9/096101

[2] R. J. D. Miller , “ Femtosecond crystallography using ultrabright electron and x-ray sources: Capturing chemistry in action,” Science 343, 1108 (2014). 10.1126/science.1248488 24604195

[3] R. J. D. Miller , “ Mapping atomic motions with ultrabright electrons: The chemists' Gedanken experiment enters the lab frame,” Ann. Rev. Phys. Chem. 65, 583 (2014). 10.1146/annurev-physchem-040412-110117 24423377

[4] B. J. Siwick , J. R. Dwyer , R. E. Jordan , and R. J. D. Miller , “ An atomic-level view of melting using femtosecond electron diffraction,” Science 302, 1382 (2003). 10.1126/science.1090052 14631036

[5] H. Ihee , V. A. Lobastov , U. M. Gomez , B. M. Goodson , R. Srinivasan , C.-Y. Ruan , and A. H. Zewail , “ Direct imaging of transient molecular structures with ultrafast diffraction,” Science 291, 458 (2001). 10.1126/science.291.5503.458 11161194

[6] B. J. Siwick , J. R. Dwyer , R. E. Jordan , and R. Miller , “ Femtosecond electron diffraction studies of strongly driven structural phase transitions,” Chem. Phys. 299, 285 (2004). 10.1016/j.chemphys.2003.11.040

[7] S. Nie , X. Wang , H. Park , R. Clinite , and J. Cao , “ Measurement of the electronic Grüneisen constant using femtosecond electron diffraction,” Phys. Rev. Lett. 96, 025901 (2006). 10.1103/PhysRevLett.96.025901 16486599

[8] T. Ishikawa , S. A. Hayes , S. Keskin , G. Corthey , M. Hada , K. Pichugin , A. Marx , J. Hirscht , K. Shionuma , K. Onda , Y. Okimoto , S.-Y. Koshihara , T. Yamamoto , H. Cui , M. Nomura , Y. Oshima , M. Abdel-Jawad , R. Kato , and R. J. D. Miller , “ Direct observation of collective modes coupled to molecular orbital–driven charge transfer,” Science 350, 1501 (2015). 10.1126/science.aab3480 26680192

[9] M. Hada , D. Zhang , K. Pichugin , J. Hirscht , M. A. Kochman , S. A. Hayes , S. Manz , R. Y. N. Gengler , D. A. Wann , T. Seki , G. Moriena , C. A. Morrison , J. Matsuo , G. Sciaini , and R. D. Miller , “ Cold ablation driven by localized forces in alkali halides,” Nat. Commun. 5, 3863 (2014). 10.1038/ncomms4863 24835317

[10] L. Waldecker , R. Bertoni , and R. Ernstorfer , “ Compact femtosecond electron diffractometer with 100 keV electron bunches approaching the single-electron pulse duration limit,” J. Appl. Phys. 117, 044903 (2015). 10.1063/1.4906786

[11] C. Gerbig , A. Senftleben , S. Morgenstern , C. Sarpe , and T. Baumert , “ Spatio-temporal resolution studies on a highly compact ultrafast electron diffractometer,” New J. Phys. 17, 043050 (2015). 10.1088/1367-2630/17/4/043050

[12] F. M. Rudakov , J. B. Hastings , D. H. Dowell , J. F. Schmerge , and P. M. Weber , “ Megavolt electron beams for ultrafast time-resolved electron diffraction,” AIP Conf. Proc. 845, 1287 (2006). 10.1063/1.2263560

[13] J. B. Hastings , F. M. Rudakov , D. H. Dowell , J. F. Schmerge , J. D. Cardoza , J. M. Castro , S. M. Gierman , H. Loos , and P. M. Weber , “ Ultrafast time-resolved electron diffraction with megavolt electron beams,” Appl. Phys. Lett. 89, 184109 (2006). 10.1063/1.2372697

[14] J. Yang , K. Kan , N. Naruse , Y. Yoshida , K. Tanimura , and J. Urakawa , “ 100-femtosecond MeV electron source for ultrafast electron diffraction,” Radiat. Phys. Chem. 78, 1106 (2009). 10.1016/j.radphyschem.2009.05.009

[15] P. Zhu , Y. Zhu , Y. Hidaka , L. Wu , J. Cao , H. Berger , J. Geck , R. Kraus , S. Pjerov , Y. Shen , R. I. Tobey , J. P. Hill , and X. J. Wang , “ Femtosecond time-resolved MeV electron diffraction,” New J. Phys. 17, 063004 (2015). 10.1088/1367-2630/17/6/063004

[16] R. K. Li and P. Musumeci , “ Single-shot MeV transmission electron microscopy with picosecond temporal resolution,” Phys. Rev. Appl. 2, 024003 (2014). 10.1103/PhysRevApplied.2.024003

[17] D. Xiang , F. Fu , J. Zhang , X. Huang , L. Wang , X. Wang , and W. Wan , “ Accelerator-based single-shot ultrafast transmission electron microscope with picosecond temporal resolution and nanometer spatial resolution,” Nucl. Instrum. Meth. A 759, 74 (2014). 10.1016/j.nima.2014.05.068

[18] S. P. Weathersby , G. Brown , M. Centurion , T. F. Chase , R. Coffee , J. Corbett , J. P. Eichner , J. C. Frisch , A. R. Fry , M. Gühr , N. Hartmann , C. Hast , R. Hettel , R. K. Jobe , E. N. Jongewaard , J. R. Lewandowski , R. K. Li , A. M. Lindenberg , I. Makasyuk , J. E. May , D. McCormick , M. N. Nguyen , A. H. Reid , X. Shen , K. Sokolowski-Tinten , T. Vecchione , S. L. Vetter , J. Wu , J. Yang , H. A. Dürr , and X. J. Wang , “ Mega-electron-volt ultrafast electron diffraction at SLAC National Accelerator Laboratory,” Rev. Sci. Instrum. 86, 073702 (2015). 10.1063/1.4926994 26233391

[19] F. Carbone , P. Musumeci , O. Luiten , and C. Hebert , “ A perspective on novel sources of ultrashort electron and x-ray pulses,” Chem. Phys. 392, 1 (2012). 10.1016/j.chemphys.2011.10.010

[20] S. Manz , A. Casandruc , D. Zhang , Y. Zhong , R. A. Loch , A. Marx , T. Hasegawa , L. C. Liu , S. Bayesteh , H. Delsim-Hashemi , M. Hoffmann , M. Felber , M. Hachmann , F. Mayet , J. Hirscht , S. Keskin , M. Hada , S. W. Epp , K. Flottmann , and R. J. D. Miller , “ Mapping atomic motions with ultrabright electrons: towards fundamental limits in space-time resolution,” Faraday Discuss. 177, 467 (2015). 10.1039/C4FD00204K 25631530

[21] D. L. Adams , H. B. Nielsen , and M. A. Van Hove , “ Quantitative analysis of low-energy-electron diffraction: Application to Pt(111),” Phys. Rev. B 20, 4789 (1979). 10.1103/PhysRevB.20.4789

[22] K. S. Novoselov , A. K. Geim , S. V. Morozov , D. Jiang , Y. Zhang , S. V. Dubonos , I. V. Grigorieva , and A. A. Firsov , “ Electric field effect in atomically thin carbon films,” Science 306, 666 (2004). 10.1126/science.1102896 15499015

[23] V. Nicolosi , M. Chhowalla , M. G. Kanatzidis , M. S. Strano , and J. N. Coleman , “ Liquid exfoliation of layered materials,” Science 340, 1226419 (2013). 10.1126/science.1226419

[24] S. Z. Butler , S. M. Hollen , L. Cao , Y. Cui , J. A. Gupta , H. R. Gutiérrez , T. F. Heinz , S. S. Hong , J. Huang , A. F. Ismach , E. Johnston-Halperin , M. Kuno , V. V. Plashnitsa , R. D. Robinson , R. S. Ruoff , S. Salahuddin , J. Shan , L. Shi , M. G. Spencer , M. Terrones , W. Windl , and J. E. Goldberger , “ Progress, challenges, and opportunities in two-dimensional materials beyond graphene,” ACS Nano 7, 2898 (2013). 10.1021/nn400280c 23464873

[25] S. Das , J. A. Robinson , M. Dubey , H. Terrones , and M. Terrones , “ Beyond graphene: Progress in novel two-dimensional materials and van der Waals solids,” Annu. Rev. Mater. Res. 45, 1 (2015). 10.1146/annurev-matsci-070214-021034

[26] G. R. Bhimanapati , Z. Lin , V. Meunier , Y. Jung , J. Cha , S. Das , D. Xiao , Y. Son , M. S. Strano , V. R. Cooper , L. Liang , S. G. Louie , E. Ringe , W. Zhou , S. S. Kim , R. R. Naik , B. G. Sumpter , H. Terrones , F. Xia , Y. Wang , J. Zhu , D. Akinwande , N. Alem , J. A. Schuller , R. E. Schaak , M. Terrones , and J. A. Robinson , “ Recent advances in two-dimensional materials beyond graphene,” ACS Nano 9, 11509 (2015). 10.1021/acsnano.5b05556 26544756

[27] A. Gupta , T. Sakthivel , and S. Seal , “ Recent development in 2D materials beyond graphene,” Prog. Mater. Sci. 73, 44 (2015). 10.1016/j.pmatsci.2015.02.002

[28] G. Binnig , H. Rohrer , Ch. Gerber , and E. Weibel , “ Surface studies by scanning tunneling microscopy,” Phys. Rev. Lett. 49, 57 (1982). 10.1103/PhysRevLett.49.57

[29] G. Binnig , C. F. Quate , and Ch. Gerber , “ Atomic-force microscope,” Phys. Rev. Lett. 56, 930 (1986). 10.1103/PhysRevLett.56.930 10033323

[30] A. Janzen , B. Krenzer , O. Heinz , P. Zhou , D. Thien , A. Hanisch , F.-J. Meyer zu Heringdorf , D. von der Linde , and M. Horn von Hoegen , “ A pulsed electron gun for ultrafast electron diffraction at surfaces,” Rev. Sci. Instrum. 78, 013906 (2007). 10.1063/1.2431088 17503932

[31] A. Hanisch-Blicharski , A. Janzen , B. Krenzer , S. Wall , F. Klasing , A. Kalus , T. Frigge , M. Kammler , and M. H. von Hoegen , “ Ultra-fast electron diffraction at surfaces: From nanoscale heat transport to driven phase transitions,” Ultramicroscopy 127, 2 (2013). 10.1016/j.ultramic.2012.07.017 22975358

[32] M. Müller , A. Paarmann , and R. Ernstorfer , “ Femtosecond electrons probing currents and atomic structure in nanomaterials,” Nat. Commun. 5, 5292 (2014). 10.1038/ncomms6292 25358554

[33] M. Gulde , S. Schweda , G. Storeck , M. Maiti , H. K. Yu , A. M. Wodtke , S. Schäfer , and C. Ropers , “ Ultrafast low-energy electron diffraction in transmission resolves polymer/graphene superstructure dynamics,” Science 345, 200 (2014). 10.1126/science.1250658 25013072

[34] B. J. Siwick , J. R. Dwyer , R. E. Jordan , and R. J. D. Miller , “ Ultrafast electron optics: Propagation dynamics of femtosecond electron packets,” J. Appl. Phys. 92, 1643 (2002). 10.1063/1.1487437

[35] Z. Tao , H. Zhang , P. M. Duxbury , M. Berz , and C.-Y. Ruan , “ Space charge effects in ultrafast electron diffraction and imaging,” J. Appl. Phys. 111, 044316 (2012). 10.1063/1.3685747

[36] T. P. Wangler , RF Linear Accelerators ( Wiley-VCH, New York, 1997).

[37] G. H. Kassier , K. Haupt , N. Erasmus , E. G. Rohwer , and H. Schwoerer , “ Achromatic reflectron compressor design for bright pulses in femtosecond electron diffraction,” J. Appl. Phys. 105, 113111 (2009). 10.1063/1.3132834

[38] M. Gao , H. Jean-Ruel , R. R. Cooney , J. Stampe , M. de Jong , M. Harb , G. Sciaini , G. Moriena , and R. J. D. Miller , “ Full characterization of RF compressed femtosecond electron pulses using ponderomotive scattering,” Opt. Express 20, 12048 (2012). 10.1364/OE.20.012048 22714191

[39] R. P. Chatelain , V. R. Morrison , C. Godbout , and B. J. Siwick , “ Ultrafast electron diffraction with radio-frequency compressed electron pulses,” Appl. Phys. Lett. 101, 081901 (2012). 10.1063/1.4747155 25526134

[40] Y. Wang and N. Gedik , “ Electron pulse compression with a practical reflectron design for ultrafast electron diffraction,” IEEE J. Quantum Electron. 18, 140 (2012). 10.1109/JSTQE.2011.2112339

[41] Y. Qi , M. Pei , D. Qi , Y. Yang , T. Jia , S. Zhang , and Z. Sun , “ Realizing ultrafast electron pulse self-compression by femtosecond pulse shaping technique,” J. Phys. Chem. Lett. 6, 3867 (2015). 10.1021/acs.jpclett.5b01305 26722884

[42] R. Brogle , P. Muggli , P. Davis , G. Hairapetian , and C. Joshi , “ Studies of linear and nonlinear photoelectric emission for advanced accelerator applications,” in *Proceedings of the 1995 Particle Accelerator Conference* (1995), vol. 2, p. 1039.

[43] S. Tsujino , P. Beaud , E. Kirk , T. Vogel , H. Sehr , J. Gobrecht , and A. Wrulich , “ Ultrafast electron emission from metallic nanotip arrays induced by near infrared femtosecond laser pulses,” Appl. Phys. Lett. 92, 193501 (2008). 10.1063/1.2924290

[44] A. Paarmann , M. Gulde , M. Müller , S. Schäfer , S. Schweda , M. Maiti , C. Xu , T. Hohage , F. Schenk , C. Ropers , and R. Ernstorfer , “ Coherent femtosecond low-energy single-electron pulses for time-resolved diffraction and imaging: A numerical study,” J. Appl. Phys. 112, 113109 (2012). 10.1063/1.4768204

[45] M. E. Swanwick , P. D. Keathley , A. Fallahi , P. R. Krogen , G. Laurent , J. Moses , F. X. Kärtner , and L. F. Velásquez-García , “ Nanostructured ultrafast silicon-tip optical field-emitter arrays,” Nano Lett. 14, 5035 (2014). 10.1021/nl501589j 25075552

[46] D. Ehberger , J. Hammer , M. Eisele , M. Krüger , J. Noe , A. Högele , and P. Hommelhoff , “ Highly coherent electron beam from a laser-triggered tungsten needle tip,” Phys. Rev. Lett. 114, 227601 (2015). 10.1103/PhysRevLett.114.227601 26196645

[47] A. Casandruc , G. Kassier , H. Zia , R. Bücker , and R. J. D. Miller , “ Fiber tip-based electron source,” J. Vac. Sci. Technol. B 33, 03C101 (2015). 10.1116/1.4902016

[48] S. Humphries, Jr. , Principles of Charged Particle Acceleration ( Wiley-Interscience, New York, 1986).

[49] L. Veisz , G. Kurkin , K. Chernov , V. Tarnetsky , A. Apolonski , F. Krausz , and E. Fill , “ Hybrid dc-ac electron gun for fs-electron pulse generation,” New J. Phys. 9, 451 (2007). 10.1088/1367-2630/9/12/451

[50] W. E. King , G. H. Campbell , A. Frank , B. Reed , J. F. Schmerge , B. J. Siwick , B. C. Stuart , and P. M. Weber , “ Ultrafast electron microscopy in materials science, biology, and chemistry,” J. Appl. Phys. 97, 111101 (2005). 10.1063/1.1927699

[51] J. R. Dwyer , R. E. Jordan , C. T. Hebeisen , M. Harb , R. Ernstorfer , T. Dartigalongue , and R. J. D. Miller , “ Femtosecond electron diffraction: an atomic perspective of condensed phase dynamics,” J. Mod. Opt. 54, 905 (2007). 10.1080/09500340601095348

[52] M. W. van Mourik , W. J. Engelen , E. J. D. Vredenbregt , and O. J. Luiten , “ Ultrafast electron diffraction using an ultracold source,” Struct. Dyn. 1, 034302 (2014). 10.1063/1.4882074 26798777 PMC4711599

[53] A. Fasolino , J. H. Los , and M. I. Katsnelson , “ Intrinsic ripples in graphene,” Nat. Mater. 6, 858 (2007). 10.1038/nmat2011 17891144

[54] J. C. Meyer , A. K. Geim , M. I. Katsnelson , K. S. Novoselov , T. J. Booth , and S. Roth , “ The structure of suspended graphene sheets,” Nature 446, 60 (2007). 10.1038/nature05545 17330039

[55] J. Brivio , D. T. L. Alexander , and A. Kis , “ Ripples and layers in ultrathin MoS_2_ membranes,” Nano Lett. 11, 5148 (2011). 10.1021/nl2022288 22010987

[56] F. O. Kirchner , S. Lahme , F. Krausz , and P. Baum , “ Coherence of femtosecond single electrons exceeds biomolecular dimensions,” New J. Phys. 15, 063021 (2013). 10.1088/1367-2630/15/6/063021

[57] J. Cao , Z. Hao , H. Park , C. Tao , D. Kau , and L. Blaszczyk , “ Femtosecond electron diffraction for direct measurement of ultrafast atomic motions,” Appl. Phys. Lett. 83, 1044 (2003). 10.1063/1.1593831

[58] B. J. Siwick , A. A. Green , C. T. Hebeisen , and R. J. D. Miller , “ Characterization of ultrashort electron pulses by electron-laser pulse cross correlation,” Opt. Lett. 30, 1057 (2005). 10.1364/OL.30.001057 15907002

[59] C. T. Hebeisen , R. Ernstorfer , M. Harb , T. Dartigalongue , R. E. Jordan , and R. J. Dwayne Miller , “ Femtosecond electron pulse characterization using laser ponderomotive scattering,” Opt. Lett. 31, 3517 (2006). 10.1364/OL.31.003517 17099769

[60] B. Barwick , D. J. Flannigan , and A. H. Zewail , “ Photon-induced near-field electron microscopy,” Nature 462, 902 (2009). 10.1038/nature08662 20016598

[61] R. Li , W. Huang , Y. Du , L. Yan , Q. Du , J. Shi , J. Hua , H. Chen , T. Du , H. Xu , and C. Tang , “ Note: Single-shot continuously time-resolved MeV ultrafast electron diffraction,” Rev. Sci. Instrum. 81, 036110 (2010). 10.1063/1.3361196 20370233

[62] M. Eichberger , N. Erasmus , K. Haupt , G. Kassier , A. v. Flotow , J. Demsar , and H. Schwoerer , “ Femtosecond streaking of electron diffraction patterns to study structural dynamics in crystalline matter,” Appl. Phys. Lett. 102, 121106 (2013). 10.1063/1.4798518

[63] F. O. Kirchner , A. Gliserin , F. Krausz , and P. Baum , “ Laser streaking of free electrons at 25 keV,” Nat. Photon. 8, 52 (2014). 10.1038/nphoton.2013.315

[64] K. Flöttmann , “ ASTRA,” http://www.desy.de/~mpyflo/ (2000).

[65] D. H. Dowell and J. F. Schmerge , “ Quantum efficiency and thermal emittance of metal photocathodes,” Phys. Rev. ST Accel. Beams 12, 074201 (2009). 10.1103/PhysRevSTAB.12.074201

[66] AS-Photonics, “ SNLO,” http://www.as-photonics.com/snlo (2015).

[67] M. Aidelsburger , F. O. Kirchner , F. Krausz , and P. Baum , “ Single-electron pulses for ultrafast diffraction,” Proc. Natl. Acad. Sci. U.S.A. 107, 19714 (2010). 10.1073/pnas.1010165107 21041681 PMC2993426

[68] P. Baum and A. H. Zewail , “ 4D attosecond imaging with free electrons: Diffraction methods and potential applications,” Chem. Phys. 366, 2 (2009). 10.1016/j.chemphys.2009.07.013

[69] H. Park and J. M. Zuo , “ Direct measurement of transient electric fields induced by ultrafast pulsed laser irradiation of silicon,” Appl. Phys. Lett. 94, 251103 (2009). 10.1063/1.3157270

[70] S. Schäfer , W. Liang , and A. H. Zewail , “ Structural dynamics and transient electric-field effects in ultrafast electron diffraction from surfaces,” Chem. Phys. Lett. 493, 11 (2010). 10.1016/j.cplett.2010.04.049

[71] P. Zhu , Z. Zhang , L. Chen , J. Zheng , R. Li , W. Wang , J. Li , X. Wang , J. Cao , D. Qian , Z. Sheng , and J. Zhang , “ Four-dimensional imaging of the initial stage of fast evolving plasmas,” Appl. Phys. Lett. 97, 211501 (2010). 10.1063/1.3521387

[72] R.-Z. Li , P. Zhu , L. Chen , J. Chen , J. Cao , Z.-M. Sheng , and J. Zhang , “ Simultaneous investigation of ultrafast structural dynamics and transient electric field by sub-picosecond electron pulses,” J. Appl. Phys. 115, 183507 (2014). 10.1063/1.4875659

[73] P. F. Zhu , Z. C. Zhang , L. Chen , R. Z. Li , J. J. Li , X. Wang , J. M. Cao , Z. M. Sheng , and J. Zhang , “ Ultrashort electron pulses as a four-dimensional diagnosis of plasma dynamics,” Rev. Sci. Instrum. 81, 103505 (2010). 10.1063/1.3491994 21034089

[74] R.-Z. Li , P. Zhu , L. Chen , T. Xu , J. Chen , J. Cao , Z.-M. Sheng , and J. Zhang , “ Investigation of transient surface electric field induced by femtosecond laser irradiation of aluminum,” New J. Phys. 16, 103013 (2014). 10.1088/1367-2630/16/10/103013

[75] R. K. Raman , Z. Tao , T.-R. Han , and C.-Y. Ruan , “ Ultrafast imaging of photoelectron packets generated from graphite surface,” Appl. Phys. Lett. 95, 181108 (2009). 10.1063/1.3259779

[76] C. M. Scoby , R. K. Li , and P. Musumeci , “ Effect of an ultrafast laser induced plasma on a relativistic electron beam to determine temporal overlap in pump-probe experiments,” Ultramicroscopy 127, 14 (2013). 10.1016/j.ultramic.2012.07.015 22951263

[77] H. Park , Z. Hao , X. Wang , S. Nie , R. Clinite , and J. Cao , “ Synchronization of femtosecond laser and electron pulses with subpicosecond precision,” Rev. Sci. Instrum. 76, 083905 (2005). 10.1063/1.1994922

[78] A. Dolocan , M. Hengsberger , H. J. Neff , M. Barry , C. Cirelli , T. Greber , and J. Osterwalder , “ Electron-photon pulse correlator based on space-charge effects in a metal pinhole,” Jpn. J. Appl. Phys. 45, 285 (2006).

[79] A. Jablonski , F. Salvat , and C. J. Powell , NIST Electron Elastic-Scattering Cross-Section Database - Version 3.2 ( National Institute of Standards and Technology, Gaithersburg, MD, 2010).

[80] S. Tanuma , C. J. Powell , and D. R. Penn , “ Calculations of electron inelastic mean free paths. IX. data for 41 elemental solids over the 50 eV to 30 keV range,” Surf. Interface Anal. 43, 689 (2011). 10.1002/sia.3522

[81] See http://www.rsc.org/periodic-table for Royal Society of Chemistry, “Periodic Table” (2015).

